# Delayed diagnosis and management of adult Hirschsprung's disease: Case report and literature review

**DOI:** 10.1016/j.ijscr.2025.110908

**Published:** 2025-01-20

**Authors:** Iyad Al Jada, Maaweya Jabareen, Wasef Alhroub, Rosol Iwaiwi, Mohammad Zeidan, Thkra Meshal

**Affiliations:** a Facility of Medicine, Hebron University, Hebron, Palestine; b Facility of Medicine, Palestine Polytechnic University, Hebron, Palestine

**Keywords:** Hirschsprung's disease, Adult presentation, Chronic constipation, Post-operative diagnosis, Literature review

## Abstract

**Background:**

Hirschsprung's disease (HD) is a congenital disorder that is primarily diagnosed in infancy. Adult presentations are extremely rare and often result in delayed diagnosis and management challenges.

**Case presentation:**

A 27-year-old male with a history of chronic constipation presented with acute bowel obstruction after two weeks of failed stool passage despite repeated enemas. Physical examination revealed abdominal distension and absent bowel sounds. Urgent laparotomy demonstrated significant colonic dilation extending from the rectum to the cecum. A panproctocolectomy with ileostomy was performed, and postoperative biopsy confirmed HD. The patient recovered well and was discharged five days postoperatively.

**Discussion:**

Adult-onset HD is challenging to diagnose due to its atypical presentations. A review of 27 adult cases reveals that chronic constipation and obstruction are common symptoms, underscoring the importance of considering HD in similar presentations to prevent severe complications such as toxic megacolon and volvulus.

**Conclusion:**

This case highlights the need for high suspicion of HD in adults with chronic constipation and obstruction. Early diagnosis and intervention can significantly improve outcomes and prevent complications.

## Highlights

• Hirschsprung's disease is a congenital condition caused by the absence of nerve ganglion cells.
• Hirschsprung's disease is rarely identified in adults, leading to diagnostic challenges.
• Delayed diagnosis of Hirschsprung's disease can result in severe complications.
• Hirschsprung's disease should be considered as a differential diagnosis in cases of chronic constipation.

## 1 Introduction

This work has been reported in line with the SCARE criteria [1].

Hirschsprung's disease (HD), also known as congenital aganglionic megacolon, is a congenital disorder characterized by the absence of ganglion cells in the distal bowel, leading to functional obstruction and chronic constipation. The condition is typically diagnosed in infancy or early childhood, with an incidence of approximately 1 in 5000 live births. It predominantly affects males, with a male-to-female ratio of 4:1 [2].

HD is characterized by the absence of ganglia in the distal colon and rectum, which disrupts bowel peristalsis, leading to fecal accumulation and colonic dilation upstream. It is typically diagnosed early in life based on symptoms such as delayed meconium passage, abdominal distension, and failure to thrive. The diagnosis is confirmed through a rectal biopsy, which reveals the absence of ganglion cells, and anal manometry, which demonstrates a lack of the normal inhibitory reflex [3].

However, in rare cases, HD is not diagnosed until adulthood. Adult cases are particularly challenging because the symptoms are often subtle or atypical, leading to delays in diagnosis and management. In adults, the disease may present as severe chronic constipation, abdominal pain, or intestinal obstruction. This delayed diagnosis can result in serious complications, including toxic megacolon, sigmoid volvulus, and bowel perforation [4].

This case report details the presentation and management of a 27-year-old male patient who was first evaluated at our private center. His case underscores the critical importance of considering HD in adults presenting with severe constipation and related complications. The patient's journey through diagnosis and treatment highlights both the challenges and the necessity of timely and accurate identification of this rare condition.

## 2 Case presentation

**Fig. 1:**
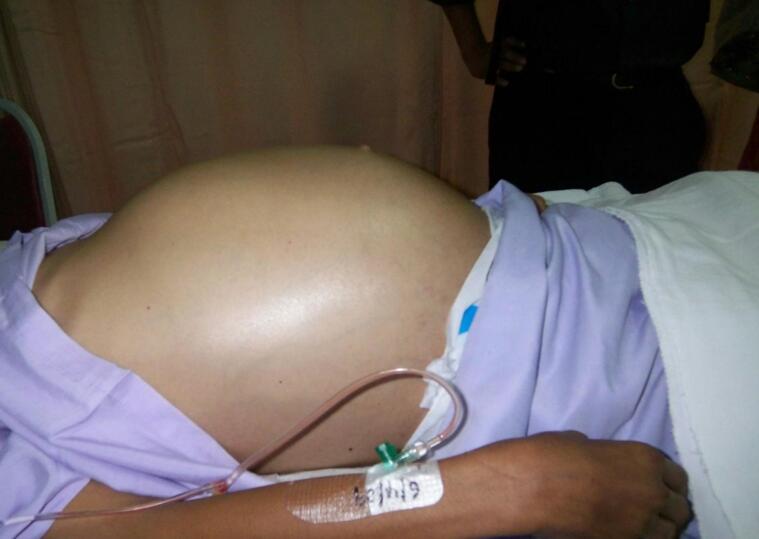
Image showing the degree of abdominal distention.

A 27-year-old male with a history of severe constipation since childhood, intermittently managed with enemas and laxatives, presented to our hospital after two weeks of failed stool passage despite repeated enemas. On admission, he exhibited signs of dehydration and tachycardia, with vital signs as follows: blood pressure, 98/60 mmHg; heart rate, 120 beats/min; respiratory rate, 18 breaths/min; temperature, 37.0 °C; and oxygen saturation, 97 % on room air. Physical examination revealed a grossly distended, non-tender abdomen with absent bowel sounds (Fig. 1). Laboratory tests were normal.

**Fig. 2:**
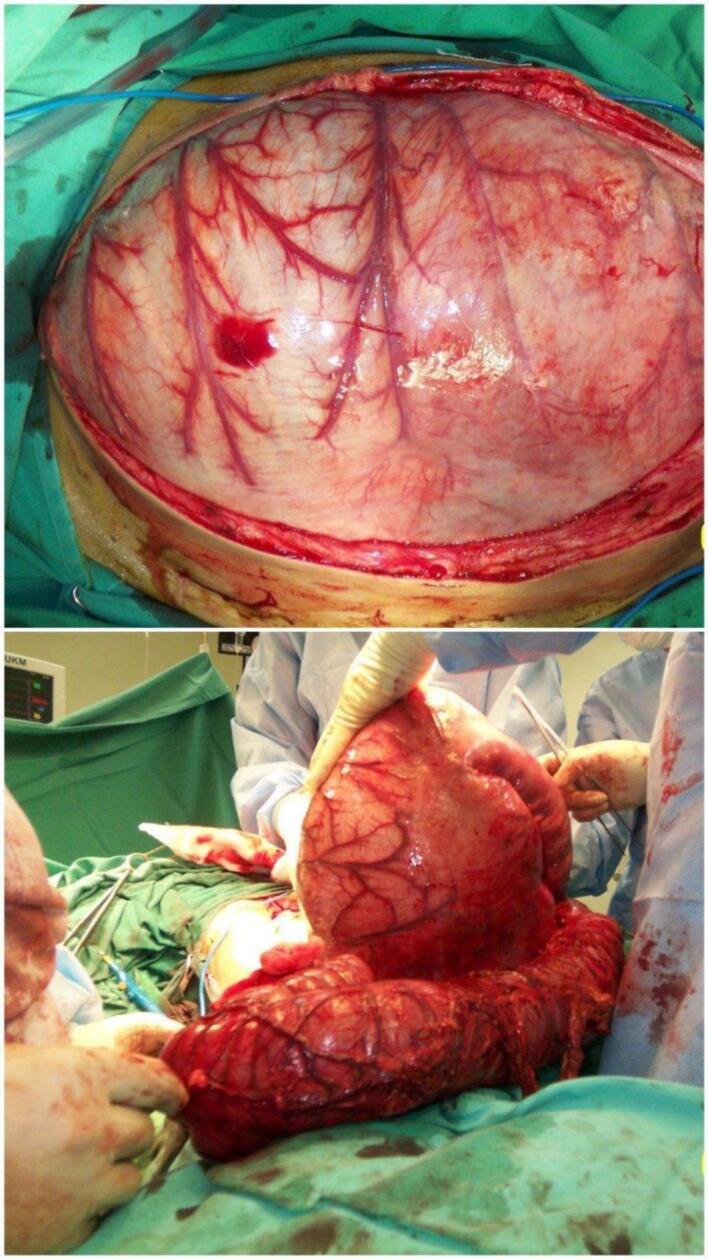
Images showing the degree of toxic megacolon.

**Fig. 3:**
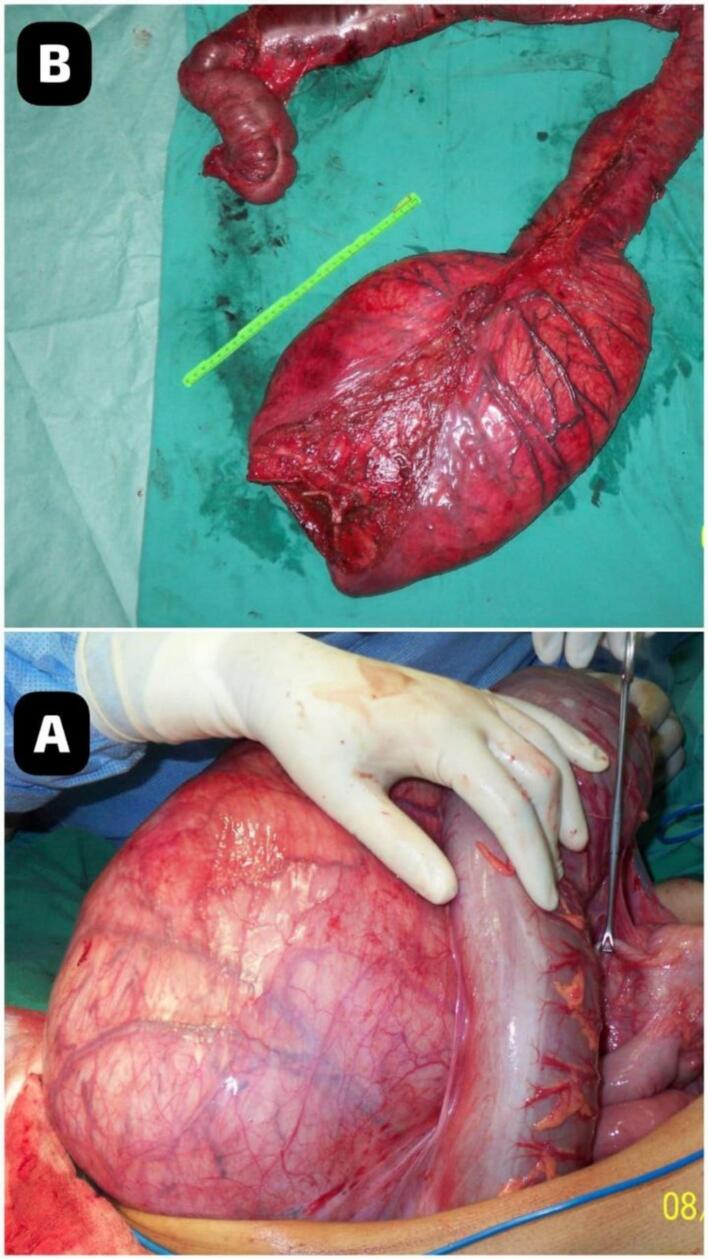
images showing A. sever extended dilated colon B. resected part of the intestines.

Due to the patient's hemodynamic instability and extensive colonic dilation, an urgent laparotomy was performed. A midline abdominal incision was made, and the abdomen was explored to assess the extent of the dilation. The surgeon observed a markedly dilated colon extending from the rectum to the cecum, with severe distension (Fig. 2) and (Fig. 3). Following this, panproctocolectomy with ileostomy was performed.

Postoperatively, the patient was closely monitored in the ICU with comprehensive care, including pain management, fluid and electrolyte balance, wound and stoma care, and vital sign monitoring. A postoperative rectal biopsy confirmed HD. The patient was discharged after five days following stabilization and improvement in his condition.

## 3 Discussion and review

HD is a condition marked by the absence of ganglion cells in the submucosal (Meissner's) and myenteric (Auerbach's) plexuses within the lower gastrointestinal tract [5]. While most cases of HD are diagnosed in childhood, typically before the age of one, there are rare instances where the diagnosis is made in adulthood [5,6]. In 1886, Harald Hirschsprung first identified Hirschsprung disease (HSCR) in older children [8]. While the first documented case of HD in an adult was reported by Rosin and colleagues in 1951 [6].

The clinical presentation of HD in adults is predominantly linked to chronic constipation. A literature review conducted in 2021 found that constipation was a symptom in all the cases examined in the study. Other symptoms like abdominal pain, abdominal distension, and fecal impaction can occur [9].

Diagnosis of HD is most accurately confirmed through rectal aspiration biopsy, which detects the absence of ganglion cells using histochemical staining [10]. Additionally, abdominal imaging, barium enema, and anorectal manometry can provide valuable diagnostic information [10,11]. Anorectal manometry typically reveals the absence of the normal anorectal inhibitory reflex [13]. Traditionally, a barium enema shows a narrowed distal colon with proximal dilatation [14], but this classic finding may be less apparent in adults due to the chronic nature of the disease, which can lead to rectal fecal impaction.

HD may remain undiagnosed until acute complications arise, such as toxic megacolon, sigmoid volvulus, and bowel perforation [4]. Kusuma et al. documented a case where adult HD led to intestinal obstruction caused by volvulus [15]. Anadani et al. reported another adult case where signs of peritonitis were found, leading to the discovery of intestinal perforation due to obstruction [16]. Additionally, Joseph et al. described a case with a highly dilated rectum and extensive necrosis at the base [17].

The definitive management of HD is surgical, with several techniques offering distinct approaches and considerations. The Duhamel procedure involves mobilizing the ganglionic segment of the colon through the posterior wall of the rectum to the anal canal, where it is connected with a crushing clamp, preserving the aganglionic rectum as a reservoir to minimize recurrence risk [18]. The Swenson technique involves resecting the sigmoid and rectum, removing both the aganglionic segment and the dilated proximal colon. Following this, a coloanal anastomosis is created by everting the rectal stump through the anus [19]. The modified Soave procedure, adapted from Faucheron and Jarry's approach, involves laparoscopic rectosigmoid resection combined with transanal colonic pull-through and delayed coloanal anastomosis without a protective stoma. This minimally invasive technique is favored for reducing deep pelvic tissue damage and enhancing precision through better visibility [20]. Rehbein's procedure for HD aims to address the condition by excising the enlarged sigmoid colon and the aganglionic segment. This is achieved through a deep dissection of the upper rectum into the pelvic cavity, around 2 cm from the peritoneal reflection. The procedure also involves treating internal anal sphincter achalasia through aggressive dilation [22].

We present a severe case of adult HD, diagnosed postoperatively and showing extensive megacolon. The patient, who had presented with chronic constipation since childhood, was successfully managed with a panproctocolectomy and ileostomy.

In this review, based on our research, we analyzed 27 case reports published since 1978 and summarized in (Table 1) to compare various aspects of HD in patients older than 10 years. We included all relevant English-language cases of adult HD involving both sexes. Our goal was to evaluate differences and similarities across these cases regarding patient history, symptoms, age, surgical interventions, and outcomes.

**Table 1:** Summary of published Adult Hirschsprung's disease cases from 1978 to 2024.

Title	Date of publication	Reference
Hirschsprung's disease in adults: Report of a case and review of the literature	1978	[1]
Chronic constipation and abdominal distension in a patient with adult Hirschprung's disease and bilateral ovarian teratomas	2004	[2]
Hirschsprung's disease in adults: report of a case and review of the literature	2005	[3]
Hirschsprung's disease in a young adult: report of a case and review of the literature	2006	[4]
Adult Hirschsprung's disease presenting as sigmoid volvulus: a case report and review of literature	2006	[5]
Clinical features' diagnostics and treatment of Hirschsprung's disease in adults	2010	[6]
Hirschprung's disease in adults		[7]
[Alertness is its own reward: adult type Hirschprung's disease–clinical description of first two cases in Israel]	2012	[8]
Adult Hirschsprung's disease: report of four cases	2013	[9]
Adulthood hirschprung's disease: a report of 4 cases in Ile-Ife. Nigeria	2013	[10]
Adult Hirschsprung's disease	2015	[11]
Hirschsprung's disease in adults: the Duhamel procedure	2016	[12]
Hirschsprung disease with debut in adult age as acute intestinal obstruction: case report	2016	[13]
Hirschsprungs disease in adults two case reports and review of the literature	2018	[14]
Hirschsprung disease in an adult with intestinal malrotation and volvulus: an exceptional association		[15]
Diagnosis and surgical approach of adult Hirschsprung's disease: About two observations and review of the literature. Case series	2019	[16]
Hirschsprung's Disease: A Rare Adult Diagnosis	2020	[17]
Hirschsprung's Disease in an Adult		[18]
Adults Hirschsprung's disease, a call for awareness. A Case Report and review of the literature	2021	[19]
Adult Hirschsprung's disease: A case report and literature review	2021	[20]
A case report of Hirschsprung's disease presenting as sigmoid volvulus and literature review, Tikur Anbessa Specialized Hospital, Addis Ababa, Ethiopia	2021	[21]
Hirschsprung's Disease in Adults, A Case Report	2021	[22]
Adult Hirschsprung disease as acute intestinal obstruction: a case report		[23]
Adult Hirschprung's disease diagnosed postoperatively: A case report	2022	[24]
Adult Hirschsprung's disease presenting as chronic constipation: a case report	2023	[25]
Suspicion diagnostic of Hirschsprung's disease in an adult intraoperatively: A case report	2024	[26]
Ultrashort segment adult Hirschsprung disease: A case report of periodic abdominal distension and constipation spanning for more than 20 years	2024	[27]

References

[1] P.P. Metzger, D.T. Alvear, G.C. Arnold, R.R. Stoner, Hirschsprung's disease in adults, Dis. *Colon Rectum* 21 (1978) 113–117. doi:10.1007/BF02586453.

[2] J. Vo, R. Hayler, A. Tyler, K. Verschuer, Chronic constipation and abdominal distension in a patient with adult Hirschprung's disease and bilateral ovarian teratomas, J. Surg. Case Reports 2024 (2024). doi:10.1093/jscr/rjae227.

[3] M. Miyamoto, K. Egami, S. Maeda, K. Ohkawa, N. Tanaka, E. Uchida, T. Tajiri, Hirschsprung's Disease in Adults: Report of a Case and Review of the Literature, J. Nippon Med. Sch. 72 (2005) 113–120. doi:10.1272/jnms.72.113.

[4] F. Chen, J.H. Winston, S.K. Jain, W.L. Frankel, Hirschsprung's disease in a young adult: report of a case and review of the literature, Ann. Diagn. Pathol. 10 (2006) 347–351. doi:10.1016/j.anndiagpath.2006.03.017.

[5] F.L.S. Tan, Y.-M. Tan, S.M. Heah, F. Seow-Choen, Adult Hirschsprung's disease presenting as sigmoid volvulus: a case report and review of literature, Tech. Coloproctol. 10 (2006) 245–248. doi:10.1007/s10151-006-0288-8.

[6] G.I. Vorobyov, S.I. Achkasov, O.M. Biryukov, Clinical features' diagnostics and treatment of Hirschsprung's disease in adults, Color. Dis. 12 (2010) 1242–1248. doi:10.1111/j.1463-1318.2009.02031.x.

[7] C. Fortea-Sanchis, D. Martínez-Ramos, I. Rivadulla-Serrano, J.M. Daroca-José, G. Paiva-Coronel, J.L. Salvador-Sanchis, Hirschprung's disease in adults, Rev. Española Enfermedades Dig. 103 (2011). doi:10.4321/S1130-01082011000300007.

[8] S. Argov, O. Levandovsky, H. Kerner, O. Ben-Yizhak, U. Klinehouse, P. Reissman, [Alertness is its own reward: adult type Hirschprung's disease–clinical description of first two cases in Israel]., Harefuah 151 (2012) 18–9, 63. https://doi.org/22670495.

[9] J.-F. Qiu, Y.-J. Shi, L. Hu, L. Fang, H.-F. Wang, M.-C. Zhang, Adult Hirsch sprung's disease: report of four cases., Int. J. Clin. Exp. Pathol. 6 (2013) 1624–30. https://doi.org/23923081.

[10] O.A. Arowolo, O.O. Lawal, A.O. Adisa, V.A. Adetiloye, A.I. Afolabi, O.A. Sowande, Adulthood hirschprung's disease: a report of 4 cases in Ile-Ife. Nigeria., Afr. J. Med. Med. Sci. 42 (2013) 277–82. https://doi.org/24579391.

[11] J.P. Martinez, Adult Hirschsprung's disease, CJEM 17 (2015) 704–705. doi:10.1017/cem.2014.57.

[12] J. Vanoorbeek, M. Kint, J.-P. Yvergneaux, Hirschsprung's Disease in Adults: the Duhamel Procedure, Acta Chir. Belg. 104 (2004) 304–308. doi:10.1080/00015458. 2004.11679559.

[13] J.A. López Ruiz, L. Tallón Aguilar, L. Sánchez Moreno, J. López Pérez, F. Pareja Ciuró, F. Oliva Mompeán, F.J. Padillo Ruiz, Hirschsprung disease with debut in adult age as acute intestinal obstruction: case report, Rev. Española Enfermedades Dig. 108 (2016). doi:10.17235/reed.2016.3841/2015.

[14] R. Škába, J. Hoch, Z. Jech, M. Kynčl, V. Campr, [Hirschsprungs disease in adults two case reports and review of the literature]., Rozhl. Chir. 97 (2018) 133–138. http://www.ncbi.nlm.nih.gov/pubmed/29589457.

[15] E. Lupon, F. Labbe, E. Nini, S. Sondji, Hirschsprung disease in an adult with intestinal malrotation and volvulus: an exceptional association, J. Med. Case Rep. 13 (2019) 124. doi:10.1186/s13256-019-2020-0.

[16] H. Adamou, I. Amadou Magagi, O. Habou, O. Adakal, M.B. Aboulaye, A. Robnodji, L. James Didier, R. Sani, H. Abarchi, Diagnosis and surgical approach of adult Hirschsprung's disease: About two observations and review of the literature. Case series, Ann. Med. Surg. 48 (2019) 59–64. doi:10.1016/j.amsu. 2019.10.017.

[17] K. Schmutz, G. McGaig, B.J. Theiling, Hirschsprung's Disease: A Rare Adult Diagnosis, Clin. Pract. Cases Emerg. Med. 4 (2020) 480–481. doi:10.5811/cpcem.2020.6.46492.

[18] K.A. Shair, E. Edwards, Hirschsprung's Disease in an Adult, Am. J. Med. 133 (2020) e622–e624. doi:10.1016/j.amjmed.2020.02.022.

[19] C.O. Reategui, C.A. Spears, G.A. Allred, Adults Hirschsprung's disease, a call for awareness. A Case Report and review of the literature, Int. J. Surg. Case Rep. 79 (2021) 496–502. doi:10.1016/j.ijscr.2021.01.090.

[20] C. Gamez, T.O. de Boer, N. Saca, L. Umbu, S. Shoukry, P. Mashburn, P.M. DeVito, Adult Hirschsprung's disease: A case report and literature review, Int. J. Surg. Case Rep. 82 (2021) 105881. doi:10.1016/j.ijscr.2021.105881.

[21] A.W. Gosaye, T.S. Nane, T.M. Negussie, A case report of Hirschsprung's disease presenting as sigmoid volvulus and literature review, Tikur Anbessa Specialized Hospital, Addis Ababa, Ethiopia, BMC Surg. 21 (2021) 109. doi:10.1186/s12893-020-00938-x.

[22] M. Haida, H. Ouaya, M. Michouar, A.A. Errami, S. Oubaha, Z. Samlani, K. Krati, Hirschsprung's Disease in Adults, A Case Report, Sch. J. Appl. Med. Sci. 9 (2021) 1427–1430. doi:10.36347/sjams.2021.v09i09.022.

[23] M.I. Kusuma, S. Sampetoding, M. Bahrun, M. Faruk, Adult Hirschsprung disease as acute intestinal obstruction: a case report, Pan Afr. Med. J. 41 (2022). doi:10.11604/pamj.2022. 41.11.31148.

[24] R. Anadani, A. Aladna, S. Radwan, Z. Zeino, F. Albakkar, A. Aljaber, N. Mahli, Adult Hirschprung's disease diagnosed postoperatively: A case report, Ann. Med. Surg. 76 (2022). doi:10.1016/j.amsu.2022.103512.

[25] T.M. Rahardjo, Y.A. Nurzaman, J. Natalia, I. Hapdijaya, L. Devina, H. Andrianto, J.C. Mahardhika, Adult Hirschsprung's disease presenting as chronic constipation: a case report, J. Med. Case Rep. 17 (2023) 308. doi:10.1186/s13256-023-03986-y.

[26] T. Joseph, A. Jean Paul, A. Francis, O. Joseph, Suspicion diagnostic of Hirschsprung's disease in an adult intraoperatively: A case report, Trauma Case Reports 53 (2024) 101088. doi:10.10 16/j.tcr.2024.101088.

[27] A. Jha, H. Sapkota, P. Ghimire, N. Paudel, R. Ranabhat, S. Jha, Ultrashort segment adult Hirschsprung disease: A case report of periodic abdominal distension and constipation spanning for more than 20 years, Radiol. Case Reports 19 (2024) 5328–5331. doi:10.10 16/j.radcr.2024.08.011.

Our study encompassed 43 patients across different study types, including 20 case reports, 3 case series, 3 case reports with literature reviews, and 1 case series with a literature review. The demographic data revealed a male predominance, with 27 males compared to 16 females, yielding a male-to-female ratio of 1.7:1, and an average age of 29.8 years.

Chronic constipation and intestinal obstruction with distention were prevalent, affecting 97.6 % of patients. Fecal impaction was observed in 86 % of cases, and pain was reported in 41.8 % of patients. Symptom management attempts included laxatives or home remedies used by 48.8 % of the cases. Nausea and vomiting were less common, affecting 18.6 %, and malnutrition was reported in 20.9 % of cases. Sigmoid volvulus was present in 18.6 % of patients, and delayed meconium was noted in 9.3 %.

Diagnostic methods included barium enemas performed on 69.7 % of patients, biopsies conducted on 83.7 %, and anorectal manometry used in 9.3 % of cases. Other imaging techniques were employed by 23.2 % of the physicians. Management strategies varied, with Duhamel's procedure used in 20.9 % of cases (one case with complications, 11.1 %), Soave's procedure in 20.9 % of cases (one case with a surgical complication, 11.1 %), Swenson's procedure in 13.9 % (two cases with complications, 33.3 %), and Rehbein's procedure performed in 6.9 % of cases. Additionally, 9.3 % of patients received other treatments, with three cases involving complications (75 %). In 6.9 % of cases, conservative management was provided (one case with complications).

Given the high prevalence of chronic constipation, intestinal obstruction, and fecal impaction in these cases—affecting 97.6 % and 86 % of patients, respectively—clinicians should consider HD in the differential diagnosis for adults presenting with these symptoms. The significant percentage of patients with these gastrointestinal issues suggests that HD should be evaluated as a potential underlying condition in adults with severe and persistent symptoms. This approach could lead to earlier diagnosis and more effective management, ultimately improving patient outcomes.

## 4 Conclusion

HD, typically diagnosed in childhood, can also present in adults, often leading to delayed diagnosis and complex management. This case report and literature review emphasizes the need to consider HD in adults with severe chronic constipation and gastrointestinal complications. Early diagnosis is crucial to preventing severe outcomes such as intestinal obstruction and volvulus.

This case of a 27-year-old male required a panproctocolectomy with ileostomy, demonstrating the benefits of timely surgical intervention. The review further underscores the importance of a comprehensive diagnostic approach including rectal biopsy, anal manometry, and imaging and maintaining a high index of suspicion among healthcare providers. Recognizing HD in adults can significantly improve diagnosis and treatment outcomes, enhancing patient prognosis and quality of life. Further research is needed to better assess the prevalence of HD worldwide, which will aid in understanding its impact and improving management strategies.

## Author contribution

Maaweya Jabareen handled conceptualization, data curation, and the writing of the original draft. Wasef Alhroub handeld review, editing, and software. Rosol Iwaiwi and Thkra Meshal contributed to the investigation and visualization. Mohammad Zeidan managed resources and validation. Iyad Al Jada provided supervision.

## Informed consent

Written informed consent was obtained from the patient for the publication of this case report and its accompanying images. A copy of the consent form is available for review by the Editor-in-Chief upon request.

## Guarantor

Iyad Al jada is the guarantor for this study, taking full responsibility for the research and its outcomes. Iyad Al jada had access to all the data and made the final decision to publish the study.

## Research registration number

None.

## Funding

This research did not receive any specific grants from funding agencies in the public, commercial, or not-for-profit sectors.

## Ethic approval

Ethical approval was not applicable for this study, as our institution's IRB committee at Hebron University does not mandate approval for reporting individual cases or case series.

## Conflict of interest statement

There is no conflict of interest.

## Data Availability

All data supporting the study's findings are included in the article and are readily accessible.
